# Impact of socioeconomic status on end-of-life costs: a systematic review and meta-analysis

**DOI:** 10.1186/s12904-020-0538-y

**Published:** 2020-03-23

**Authors:** Caberry W. Yu, S. Mohammad Alavinia, David A. Alter

**Affiliations:** 1 School of Medicine, Faculty of Health Sciences, Queen’s University, 15 Arch St, Kingston, ON K7L 3N6 Canada; 2 Neural Engineering & Therapeutics Team, Toronto Rehabilitation Institute, University Health Network, 550 University Ave, Toronto, Canada; 3 Division of Physical Medicine and Rehabilitation, Department of Medicine, University of Toronto, 27 King’s College Cir, Toronto, Canada; 4 Department of Medicine, University Health Network, 27 King’s College Cir, Toronto, ON M5S 1A1 Canada; 5 Institute of Health Policy, Management and Evaluation (IHPME), University of Toronto, 4th Floor, 155 College St, Toronto, ON M5T 3M6 Canada; 6 Faculty of Medicine, University of Toronto, Medical Sciences Building, 1 King’s College Cir, Toronto, ON M5S 1A8 Canada; 7 Cardiac Rehabilitation and Secondary Prevention Program, Toronto Rehabilitation Institute, University Health Network, University of Toronto, 550 University Ave, Toronto, ON M5G 2A2 Canada; 8 IC/ES (Institute for Clinical Evaluative Sciences), 2075 Bayview Avenue, G1-06, Toronto, Ontario M4N 3M5 Canada

**Keywords:** End-of-life, Palliative, Social determinants, Income, Healthcare costs

## Abstract

**Background:**

Socioeconomic inequalities in access to, and utilization of medical care have been shown in many jurisdictions. However, the extent to which they exist at end-of-life (EOL) remains unclear.

**Methods:**

Studies in MEDLINE, EMBASE, CINAHL, ProQuest, Web of Science, Web of Knowledge, and OpenGrey databases were searched through December 2019 with hand-searching of in-text citations. No publication date or language limitations were set. Studies assessing SES (e.g. income) in adults, correlated to EOL costs in last year(s) or month(s) of life were selected. Two independent reviewers performed data abstraction and quality assessment, with inconsistencies resolved by consensus.

**Results:**

A total of twenty articles met eligibility criteria. Two meta-analyses were performed on studies that examined total costs in last year of life – the first examined costs without adjustments for confounders (*n* = 4), the second examined costs that adjusted for confounders, including comorbidities (*n* = 2). Among studies which did not adjust for comorbidities, SES was positively correlated with EOL costs (standardized mean difference, 0.13 [95% confidence interval, 0.03 to 0.24]). However, among studies adjusting for comorbidities, SES was inversely correlated with EOL expenditures (regression coefficient, −$150.94 [95% confidence interval, −$177.69 to -$124.19], 2015 United States Dollars (USD)). Higher ambulatory care and drug expenditure were consistently found among higher SES patients irrespective of whether or not comorbidity adjustment was employed.

**Conclusion:**

Overall, an inequality leading to higher end-of-life expenditure for higher SES patients existed to varying extents, even within countries providing universal health care, with greatest differences seen for outpatient and prescription drug costs. The magnitude and directionality of the relationship in part depended on whether comorbidity risk-adjustment methodology was employed.

## Background

Over the last century, developed nations had made significant improvements in overall health outcomes and life expectancy. Health care equity was a primary motivation for universal health insurance systems; however, socioeconomic status (SES) has remained a determinant of health and has strong associations with morbidity and mortality [1, 2]. Patients with low SES die younger, have higher disease burden, use less preventative health, and present later in the disease course [3, 4]. Low SES patients also have greater health care needs compared to the general health care population [5]. As SES is a multifaceted phenomenon, the measurement and definition of SES is complex and can range from income, education, and occupation, to harder to measure variables such as health behaviour, social participation and composite measures such as social deprivation indices [6, 7]. In general, low SES patients have decreased access to needed health care services, even in countries with universal health coverage [8, 9].

The end-of-life (EOL) period not only represents a period of high health care use, unmeasured differences in medical need across patient populations are also thought to be attenuated in the terminal years by virtue of the fact that all such patients die. Differences in EOL expenditures according to SES may represent variations in access to medical care, and yield insights in health care seeking behaviours, location of care, medical decision-making, and health care resource allocation [10]. As health care expenditure increases rapidly in the time close to death, understanding SES inequalities at EOL is necessary for future health care planning to reduce such social inequalities [11–13]. Accordingly, the objective of this review was to evaluate the relationship between SES and cost of health care at EOL.

## Methods

### Data sources and searches

The Preferred Reporting Items for Systematic Reviews and Meta-Analyses (PRISMA) guidelines were followed in the conduct and reporting of this meta-analysis [14]. We searched the following databases for eligible studies: MEDLINE, EMBASE, CINAHL, ProQuest, Web of Science, Web of Knowledge, and OpenGrey. Searches through December 2019 were conducted with no language or publication date restrictions. The following keywords were applied to the search: (income or socioeconomic or education) AND (end-of-life or last year* of life or last month* of life) AND (cost* or expenditure* or spending*). See Additional file 1 for all strategies used. Bibliographies from relevant publications were checked to identify relevant articles.

### Eligibility criteria

For the qualitative analysis, we included studies that were observational studies with a sample size of over 1000 adult (18 years or older) participants to reduce sampling error, as studies often compared ≥2 socioeconomic groups. No language, publication date, or publication status restrictions were imposed. Each study had a measure of SES, including income (individual, household, or neighbourhood), education, or other proxy measures such as neighbourhood poverty rate. Notably, studies that only examined race, ethnicity, or occupations in relation to EOL cost were excluded as these measures could influence EOL costs in ways beyond what can be accounted for by income [15]. The outcome measure was health-related costs in the last year(s) or month(s) of life. In addition, studies must investigate cost at EOL in relation to SES.

For the meta-analyses, we included articles selected in the systematic review that also satisfied the following conditions: the study provided EOL cost data for the last year of life, measured SES by income (individual, household, or neighbourhood), and measured total health care costs (total absolute or total covered by an insurance scheme) for a high- and a low-income category of participants. All studies included in the meta-analyses were peer-reviewed and not limited to the focus of a specific disease category (i.e. cancer).

### Study selection

Two reviewers independently screened the titles and abstracts of all references, both were blinded to the authorship (CY, DA). Potentially relevant articles were retrieved, and full texts were screened independently by the two reviewers using an eligibility checklist for inclusion in systematic review. Disagreements were resolved by consensus. All reasons for exclusion were documented into a PRISMA flowchart [14]. For all included studies, another eligibility checklist was used for inclusion in the meta-analyses.

### Data extraction and management

For study characteristics, first author, year of publication, location, purpose, study design, and funding source were extracted from included studies. For cohort characteristics, the number of patients, inclusion/exclusion criteria, and data source used to identify patients were extracted. The type of SES measurement and data sources were extracted, as well as the EOL period (e.g. last 1 year of life) and aspects of cost examined (e.g. out-of-pocket). The main outcome measures and study conclusions were summarized. It was also noted whether regression analyses were performed, and if comorbidities or clinical complexity were adjusted for.

Comprehensiveness of reporting was assessed using the Strengthening the Reporting of Observational Studies in Epidemiology (STROBE) guidelines [16]. Only studies that fulfilled ≥13 of 22 checklist items were included in the study.

For the meta-analysis, data was extracted to determine eligibility: currency used and year, EOL health care costs and/or regression results in highest and lowest SES categories in last year of life and spread measures (e.g. 95% confidence intervals).

Authors of two studies were contacted to provide unpublished EOL total costs for the meta-analysis, neither had the data required.

### Risk of bias in individual studies

Methodological quality was assessed by both reviewers independently using a modified National Heart, Lung, and Blood Institute (NHLBI) Quality Assessment Tool for Observational Cohort and Cross-Sectional Studies [17]. Eight of the 14 items of the tool were used to evaluate the risk of bias in each study, which assessed study quality within the domains of study population, data collection, and data analysis. Risk of bias categories were judged by tallying the sections’ results: > 80% yes was low risk/high quality, 60–80% yes was medium risk/medium quality, and < 60% yes was high risk/low quality.

### Synthesis of results

Two meta-analyses were performed using Review Manager (version 5.3): the first using direct cost data (no adjustments for confounders), and the second using regression results (adjusted for comorbidities) [18]. The meta-analyses were restricted to high- and medium-quality studies. All costs were converted to 2015 USD using an online tool [19].

For the meta-analysis that did not adjust for comorbidities, the principal summary measure was standardized mean difference (SMD) for the cost of last year of life between high and low SES groups. The SMD expressed the size of the intervention effect in each study relative to the variability observed in that study. An inverse-variance random-effects method was used as high levels of heterogeneity were expected due to the observational nature of studies and the variety of SES measures used [20]. For the meta-analysis that adjusted for comorbidities, we employed the use of regression coefficients for the cost of last year of life between high and low SES groups. An inverse-variance fixed-effects method was used as the heterogeneity was not significant between the studies. For both analyses, values greater than zero indicated the degree to which high SES was associated with higher EOL cost than low SES, and values less than zero indicated the degree to which high SES was associated with lower EOL cost than low SES. Regression coefficients were divided by 10 to fit the scale of the forest plot, and the interpretation of results was scaled up tenfold in response.

Heterogeneity was tested for both meta-analyses with the Breslow-Day test using method proposed by *Higgins* et al. to measure inconsistency (the percentage of total variation across studies due to heterogeneity) [21].

### Risk of bias across studies

Publication bias was assessed by evaluating a funnel plot of the effect by the inverse of its standard error. The symmetry of the plot was assessed visually. As fewer than ten studies were included in the meta-analyses, statistical tests for asymmetry were not performed [22]. We acknowledge that other factors, such as differences in study quality or true study heterogeneity, could produce asymmetry in funnel plots [23].

### Role of the funding source

This study received no specific external funding.

## Results

### Study selection

**Fig. 1:**
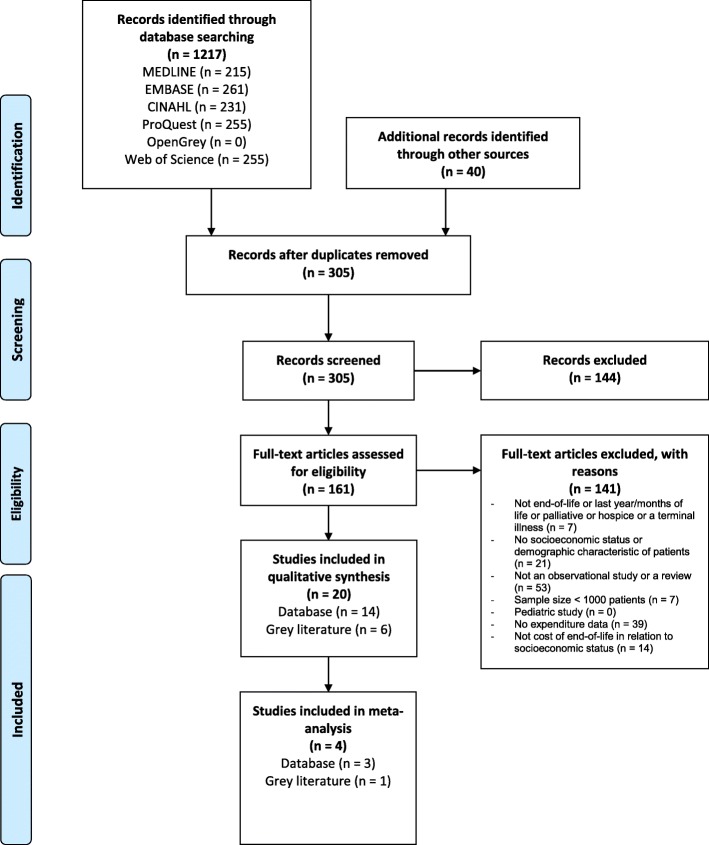
PRISMA Flowchart. The PRISMA flow diagram for the systematic review detailing the database searches, the number of abstracts screened, and the full texts retrieved

The search of databases including MEDLINE and EMBASE provided 1217 citations (Fig. 1). Additional sources yielded 40 citations. After adjusting for duplicates, 305 citations remained. Of these, 144 citations were discarded after reviewing titles and abstracts. The full texts of the remaining 161 number of studies were examined. 20 studies met the inclusion criteria and were included in the systematic review. No unpublished relevant studies were obtained.

### Study characteristics

All 20 studies selected for final review were published in English (Table 1). Seventeen were retrospective cohort studies, two were cross-sectional analyses, and one was a prospective cohort study. No existing systematic reviews were found.

**Table 1:** Summary of Studies

Source	Location	Patient Type	# of participants	SES Measured	EOL Period	Costs Measured	Results: SES-EOL Cost Relationship (Adjustment for Comorbidities)	Funding Sources Disclosed
Cunningham et al., 2011 [24]	Canada	Age 65+	58,820	Household income	Last year of life	Total medical cost	Negative relationship (adjusted); no relationship (not adjusted)	Yes
Panczak et al., 2017 [25]	Switzerland	Age 18+	113,277	Median area-based socioeconomic position index	Last year of life	Costs covered by a public health insurance	Positive relationship (not adjusted)	Yes
Kelley et al., 2015 [26]	USA	Age 70+, Medicare beneficiary	1702	Household wealth, individual education	Last 5 years of life	Total medical cost	No relationship (not adjusted)	Yes
Lee et al., 2015 [27]	Taiwan	Age 18+, oral cancer	5386	Individual income	Last month of life	Costs covered by a public health insurance	Positive but not linear relationship (adjusted)	Yes
Kelley et al., 2013 [28]	USA	Age 70+, Medicare beneficiary	3209	Individual income, individual total assets	Last 5 years of life	1 aspect of cost (out-of-pocket costs)	Positive relationship (unadjusted)	Yes
Kelley et al., 2011 [29]	USA	Age 65.5+, Medicare beneficiary	2394	Individual net worth, individual education	Last 6 months of life	Medicare costs (parts a and b)	No relationship (unadjusted)	Yes
Hanratty et al., 2007 [30]	Sweden	All decedents 18+	16,617	Household income	Last year of life	Costs covered by a public health insurance	Positive relationship (not adjusted)	Yes
Fahlman et al., 2006 [31]	USA	Age 18+, Medicare+Choice beneficiary	4602	Median area household income	Last year of life	1 aspect of cost (prescription drug costs)	Positive relationship (adjusted)	Yes
McGarry et al., 2005 [32]	USA	Age 70+	4321	Household income, household wealth	Variable (last 12 months to last 3 years of life)	1 aspect of cost (out-of-pocket costs)	Negative relationship as % of wealth (not adjusted)	Yes
Hogan et al., 2001 [33]	USA	Age 65+ or disabled, Medicare beneficiary	8000	ZIP code poverty rate	Last year of life	Medicare costs (parts a and b)	Negative relationship (not adjusted)	Yes
Timmer and Kovar, 1971 [34]	USA	Age 25+	1,649,000	Household income	Last year of life	1 aspect of cost (hospital & institutional care costs)	Positive relationship (not adjusted)	NA (government statistics)
Chen et al., 2017 [35]	Taiwan	Age 60+, Chronic Kidney Disease	65,124	Individual income	Last month of life	1 aspect of cost (inpatient costs)	Negative relationship (adjusted)	Yes
Hanchate et al., 2009 [36]	USA	Age 66+, Medicare beneficiary	158,780	Median area household income	Last 6 months of life	Medicare costs (parts a and b)	No relationship (adjusted)	Yes
Keating et al., 2018 [37]	USA	Age 65+, lung or colorectal advanced stage cancer, Medicare beneficiary	1132	Household income, individual education	Last month of life	Medicare costs (parts a and b)	No relationship (adjusted)	
Shugarman et al., 2004 [38]	USA	Age 68+, Medicare beneficiary, white or black race	241,047	Median area household income	Last 3 years of life, or year of life	Medicare costs (parts a and b)	Negative relationship for last year of life (adjusted). Positive relationship for last year of life (unadjusted). Positive relationship for last 3 years of life (regardless of adjustment)	No
Tanuseputro et al., 2015 [11]	Canada	All decedents 18+	264,755	Average area household income	Last year of life	Costs covered by a public health insurance	No relationship (unadjusted)	Yes
Rolden et al., 2014 [39]	The Netherlands	Age 65+	2833	Score based on area education and income	Last 6 months of life	Total medical cost	No relationship (unadjusted)	No
Murthy et al., 2017 [40]	Canada	All decedents 18+	264,754	Median area household income	Last year of life	Costs covered by a public health insurance	No relationship (adjusted)	Yes
Walsh & Laudicella, 2017 [41]	England	Age 18+, colorectal cancer, breast cancer, prostate cancer, or lung cancer	258,837	% of area residents on government income benefits	Last 6 months of life	1 aspect of cost (hospital costs)	Negative relationship (adjusted)	Yes
Menec et al., 2004 [42]	Canada	All decedents 18+	9436	Average area household income	Last 6 months of life	Total medical cost	No relationship (unadjusted)	Yes

*USA* United States of America; *SES* Socioeconomic status; *EOL* End-of-life

### Participants

The included studies involved 3,804,082 participants in total. The main inclusion criteria entailed adult decedents (18 years or older). Fifteen of 20 studies focused on all decedents regardless of cause, while five studies examined specific groups of patients (e.g. cancer patients). Eleven studies examined older decedents (e.g. > 65) only, and give of these studies examined Medicare beneficiaries in the United States (U.S.).

### Socioeconomic indicator (or measurement)

Ten of 20 studies examined individual or household income/net worth, 10 of 20 studies examined average income or poverty rate in the decedent’s geographic area.

### Health care cost components

Duration of health care cost measurement: nine studies measured costs for last year of life (with one also measuring last three years of life), two studies for last five years of life, five studies for last six months of life, three studies for last month of life, and one study for variable amounts of time for each decedent.

Types of health care cost measurement: The costs measured varied between studies (Table 1). They ranged from total health care cost including community care such as long-term care, home care, or complex continuing care (*n* = 3), total cost without community care (*n* = 1), total cost covered by a government-funded insurance program (*n* = 10, five of which were U.S. Medicare claims). Six studies only measured one aspect of health care cost such as prescription drug, out-of-pocket expenditure, inpatient cost, or institutional cost (*n* = 6).

### Risk of Bias within studies

Using the NHLBI tool, 10 studies were categorized as having low risk of bias, nine as medium risk, and one as high risk in Table 2.

**Table 2:** Risk of Bias in Individual Studies

Criteria	Panczak et al. 2017 [25]	Hanratty et al. 2007 [30]	Shugarman et al. 2004 [38]	Tanuseputro et al. 2015 [11]	Cunningham et al. 2011 [24]	Kelley et al. 2015 [26]	Kelley et al. 2013 [28]	Kelley et al. 2011 [29]	Lee et al. 2015 [27]	Fahlman et al. 2006 [31]	McGarry et al. 2005 [32]	Hogan et al. 2001 [33]	Timmer & Kovar 1971 [34]	Chen et al. 2017 [35]	Hanchate et al. 2009 [36]	Keating et al. 2018 [37]	Rolden et al. 2014 [39]	Murthy et al. 2017 [40]	Walsh & Laudicella 2017 [41]	Menec et al. 2004 [42]
Was the research question or objective in this paper clearly stated?	Y	Y	Y	Y	Y	Y	Y	Y	Y	Y	Y	Y	Y	Y	Y	Y	Y	Y	Y	Y
Was the study population clearly specified and defined?	Y	Y	Y	Y	Y	Y	Y	Y	Y	Y	Y	Y	Y	Y	Y	Y	Y	Y	Y	Y
Was the participation rate of eligible persons at least 50%?	Y	Y	N	Y	Y	N	Y	Y	Y	Y	Y	N	Y	Y	NR	Y	N	Y	Y	Y
Were all the subjects selected from the same or similar populations (including the same time period)? Were inclusion and exclusion criteria for being in the study prespecified and applied uniformly to all participants?	Y	Y	Y	Y	Y	Y	Y	Y	Y	Y	Y	Y	Y	Y	NR	Y	Y	Y	Y	Y
Did the study examine different levels of the exposure as related to the outcome? (i.e. SES measured as a continuous variable)	Y	Y	Y	Y	Y	N	Y	Y	Y	Y	Y	Y	Y	Y	Y	Y	Y	Y	Y	Y
Were the exposure measures (independent variables) clearly defined, valid, reliable, and implemented consistently across all study participants? (i.e. individual/household SES)	N	Y	N	N	Y	Y	Y	Y	Y	N	Y	N	Y	Y	N	Y	N	N	N	N
Were the outcome measures (dependent variables) clearly defined, valid, reliable, and implemented consistently across all study participants?	Y	Y	Y	Y	Y	Y	Y	Y	N	Y	N	N	N	Y	Y	Y	Y	Y	Y	Y
Were key potential confounding variables measured and adjusted statistically for their impact on the relationship between exposure(s) and outcome(s)? (i.e. adjusted for health status)	N	N	Y	N	Y	N	N	N	Y	Y	N	N	N	Y	Y	Y	N	Y	Y	N
Quality of Study	Medium	High	Medium	Medium	High	Medium	High	High	High	High	Medium	Low	Medium	High	Medium	High	Medium	High	High	Medium

*CD* cannot be determined; *NA* Not applicable; *NR* Not reported

### Narrative synthesis of individual studies

Studies of income-related differences in cost of health care at end-of-life showed significant heterogeneity in the relationship between SES and EOL cost. This heterogeneity resulted from differences in methodology, such as which health services were examined and whether adjustment for patients’ health status (comorbidities or clinical complexity) were made, and differences in outcomes measured, such as length of EOL period and types of cost measured. The adjustments made in each study are listed in Additional file 2.

Eleven of the 20 included studies did not adjust for comorbidities. Three studies examined total EOL cost and showed no relationship between SES and total EOL care cost [26, 39, 42]. However, higher SES was associated with higher medical out-of-pocket, hospital, and institutional care cost [28, 34]. Five studies examined total costs covered by a government-funded insurance program, which excluded out-of-pocket expenditure and costs paid by private insurance, but differed in coverage for inpatient, ambulatory, drugs/devices, and continuing care depending on the jurisdiction. For studies that examined costs covered by a public insurance scheme, low SES was associated with lower EOL cost in Switzerland and Sweden [25, 30], but not in Canada [11]. For the two studies that examined U.S. Medicare claims, one found that higher neighbourhood poverty rate was associated with higher EOL costs [33], while the other found no significant relationship between net worth and EOL cost [29].

The other nine studies adjusted for comorbidities using the Charlson Comorbidity Index Score (*n* = 5), Johns Hopkins aggregated diagnostic groups (*n* = 2), or other methods (n = 2). Low SES was associated with higher total cost and also 11% higher non-emergent hospital expenditure in the last year of life, in contrast to studies that did not adjust for comorbidities [24]. Low SES patients incurred a higher total hospital cost (acute and elective) in the last six months of life largely due to higher acute rather than elective inpatient care costs [41]. They also had higher inpatient expenses in the last month of life [35]. Consistent with studies that did not adjust for comorbidities, low SES patients had 15% lower specialist expenditure [24], 25% lower mean out-of-pocket expenses [31], and ≥ 20% lower prescription drug expenditure compared to high SES patients [24, 31]. However, while low-income patients had less out-of-pocket expenditure, it represented 70.5% of their annual income, compared to 18.3% for those in the highest income quartile [32].

Five additional studies examined total costs covered by a government-funded insurance program. When adjusted for comorbidities, studies found that SES was either not associated [40] or weakly associated [27] with EOL cost covered by public insurance schemes, compared to the mostly positive relation when unadjusted. For studies examining U.S. Medicare claims, low SES was either associated with higher EOL cost [38] or not associated with EOL cost [36, 37].

### Meta-analyses

**Fig. 2:**
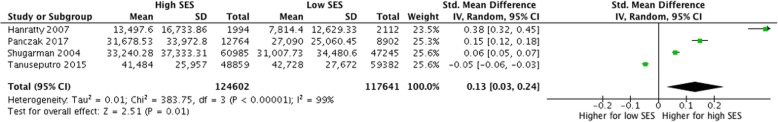
Pooled Associations between SES and EOL Cost (Unadjusted for Comorbidities). Standard mean difference (SMD) > 0 suggests that high SES is associated with higher total EOL cost in the last year of life. Diamond indicates the overall SMD with associated 95% CI. SD = standard deviation, IV = inverse variance, CI = confidence interval. Values in 2015 USD

For the meta-analysis examining the relationship between SES and EOL without adjustments for comorbidities, four studies comprising of 242,243 patients in total met the inclusion criteria. For two studies, the standard errors were estimated from reported interquartile ranges by assuming normal distributions of their costs. The four studies included in the meta-analysis which did not adjust for comorbidities all examined EOL costs covered by public insurance schemes in the last year of life – three of the four studies examined costs of government-funded health insurance that was public and universal, while one examined U.S. Medicare claims. In the pooled analysis (Fig. 2), high SES was associated with significantly higher EOL costs (SMD = 0.13, 95% CI = 0.03 to 0.24). 99% of the observed variance came from real differences between studies, rather than only random error within studies (I^2^ = 99%). As such, they can potentially be explained by study-level covariates.

**Fig. 3:**
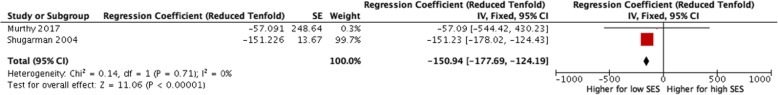
Pooled Associations Between SES and EOL Cost (Adjusted for Comorbidities). Regression coefficient > 0 suggests that high SES is associated with higher total EOL cost in the last year of life. Diamond indicates the overall regression coefficient with associated 95% CI. SE = standard error, IV = inverse variance, CI = confidence interval. Values in 2015 USD

Two studies examining the relationship between SES and EOL costs adjusting for comorbidities, comprising 505,801 patients, met meta-analysis inclusion criteria. The meta-analysis examining SES and EOL costs which did adjust for comorbidities included the same Medicare study, as well as an additional study on costs covered by a public universal insurance scheme. Standard errors were estimated from 95% CIs by assuming normal distribution. In the pooled analysis of regression coefficients adjusted for comorbidities (Fig. 3), high SES was associated with significantly lower EOL costs (regression coefficient, −$150.94 [95% CI, −$177.69 to -$124.19], 2015 USD). All observed variance came from random error within studies (I^2^ = 0%).

### Risk of Bias across studies

**Fig. 4:**
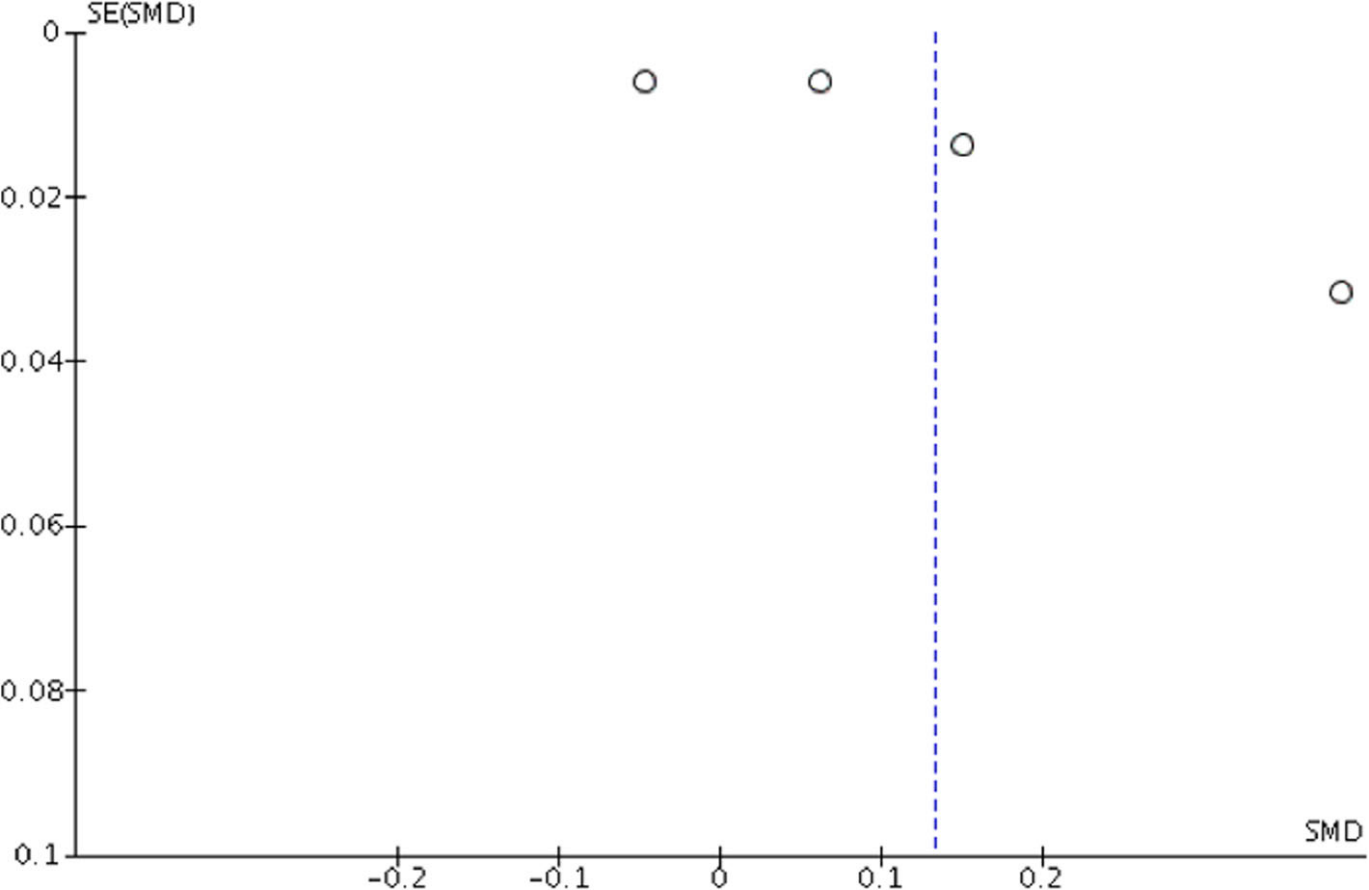
Funnel Plot for Meta-Analysis Unadjusted for Comorbidities. Funnel plot for 4 studies unadjusted for comorbidities included in meta-analysis. SMD = standard mean difference (SMD), SE = standard error

Strong evidence of heterogeneity (I^2^ = 99%, *P* < 0.0000) in the meta-analysis unadjusted for comorbidities was observed. The funnel plot showed evidence of considerable asymmetry, suggesting possibility of publication bias (Fig. 4).

### Comprehensiveness of reporting

All studies reported background/rationale, objectives, location, and key results and met 13 of the 24 STROBE criteria to be included in the review. One study did not report the data source for EOL costs. All studies reported the measures of SES used and duration of EOL cost measured. Four studies did not report the service types of EOL cost studied.

## Discussion

The aim of this review was to evaluate the relationship between SES and health expenditure at EOL. We found that patient SES was significantly correlated with EOL expenditures. Overall, the evidence suggested significant heterogeneity in units of cost, length of EOL period, extent of adjustment, and directionality of conclusions. One of the key factors that accounted for the variation in SES-EOL cost inequalities was adjustment for comorbidities. When unadjusted for comorbidities, low SES was associated with lower total and hospital EOL expenditures. Conversely, when adjusted for comorbidities, low SES was associated with higher total and hospital EOL expenditure. Irrespective of adjustment, low SES patients had lower specialist, out-of-pocket, and drug expenditure at EOL even within jurisdictions providing universal health coverage to its citizens.

To the best of our knowledge, our systematic review and meta-analyses were the first to examine the effects of socioeconomic variation on EOL cost of care. While SES has been shown to be an important determinant of population health across the life continuum [43–47], its relationship with health-care expenditures was less consistent and more variable across jurisdictions [48]. The focus on EOL care has also been less studied than the relationships between SES and health-care during other periods of life.

Our systematic review and meta-analyses demonstrated inconsistent results for the relationship between SES and total (and more specifically, hospital-related) EOL expenditures depending on whether or not individual studies employed comorbidity risk-adjustment methodology. Given the importance of comorbidity as a determinant of EOL expenditures [49, 50] and the presumed higher comorbidity disease burden among socioeconomically disadvantaged populations [24, 27, 31, 35–38, 40, 41], one would have hypothesized that SES inequalities in hospital-related EOL expenditures would have only become more pronounced among studies that incorporated comorbidity risk-adjustment than among studies that did not. Such was not the case; if anything, the converse was true. Several possibilities may explain such counterintuitive findings. First, “immortality bias” among lower SES patients who presented to hospital may have been present as a result of a disproportionately higher number of socioeconomically-disadvantaged patients with extensive comorbid disease burden succumbing from an out-of-hospital sudden cardiac death [51, 52]. Such a bias may have skewed or altered the burden of comorbidity between lower and higher SES populations. Second, comorbidity adjustment, mostly measured through Charlson Comorbidity Index Score and the Johns Hopkins Aggregated Diagnostic Groups [51, 52], may have been incomplete leading to residual unmeasured confounding. Third, unlike mortality, not all comorbidities are as predictable in their relationships with health care spending [53]. Fourth, the propensity to hospitalize patients may have been driven by factors other than comorbidities. For example, socioeconomically-disadvantaged patients may have had fewer ambulatory and community support-systems which necessitated EOL hospitalization irrespective of comorbid disease burden. Accordingly, risk-adjustment methodology may have not appropriately accounted for the health-cost consequences associated with comorbidities in general. Finally, SES itself is a complex measure that is confounded not only by comorbidity, but also by variations in psychosocial stress, social supports, health behaviours, and health literacy. Such factors when combined with medical care access inequalities, and physician medical-decision-making biases for EOL care may have in part, or together explained the inconsistencies between those studies which did and did employ risk-adjustment methodologies. Whether SES-EOL expenditure associations should employ comorbidity risk-adjustment methodology remains unclear, as comorbidity is intertwined within the SES measurement itself.

Notwithstanding the important modulating effects of comorbidity adjustments on hospital (and total) EOL expenditures, our qualitative synthesis and meta-analyses demonstrated higher EOL ambulatory care and drug expenditures among socioeconomically advantaged than disadvantaged individuals, suggesting inequity in out-of-pocket spending. While high SES patients spent more out-of-pocket in absolute amount in the last year of life [28], low SES patients had higher out-of-pocket expenditure proportional to their annual income [32]. This result extended as far back as the last five years of life [26]. In contrast, high SES patients had a higher informal caregiving cost, likely due to more availability of informal care providers and financial resources [26]. High SES patients also spent 15% more on specialist care, and 23 to 25% more on prescription drug in the last year of life than their low SES counterparts [24, 31]. Higher SES patients had more prescriptions filled and spent more money out-of-pocket on prescriptions [31]. These results were not surprising, as socioeconomic differences make accessing specialist and pharmaceuticals easier for those with higher income throughout life [54]. These differences include education, private support systems, employer-based insurance, and relative affordability of any of out-of-pocket payments [24]. It is also important to note that most studies examining out-of-pocket and drug expenditure were based in the U.S.

Reasons for SES-EOL expenditure inequalities may be multifactorial and complex. Different models of care, durations of EOL period investigated, sociocultural preferences around death and dying, and definitions of “high” vs “low” SES may be other potential explanations for between-country differences. For example, studies on total EOL costs covered by public insurance schemes varied from weak positive SES-EOL relationships for countries that provide universal public insurance [11, 25, 27, 30, 40], to weak inverse relationships for U.S. Medicare [29, 33, 36–38]. There was some evidence that factors generating the observed inequalities in universal health care systems differed from those in market-based health care systems. For example, in the Swedish study by Hanratty and colleagues, patients with high SES had a higher median number of hospital bed days [30], which contrasted the higher inpatient costs associated with low SES patients found in other studies [24, 35, 41]. This suggests a higher use of hospital care in Sweden for higher SES patients that was not present in other countries.

Future studies examining the impact of SES on EOL cost should examine costs of individual service types as well as aggregated costs, and adjust for health care status using measures of clinical complexity such as comorbidities [24]. The complexity in the associations between SES and EOL care use and cost necessitates careful examination of modulators such as location of death, cause of death, and age at death. The relationships between SES and other factors important to EOL care, such as age and race, must be explored to understand the social patterning in the terminal years of life.

Our study has important population health and health service research implications. First, the demonstration of SES-health expenditure inequalities at the EOL reinforce the notion that SES-related health and/or health-care differences persist not just at younger phases of life, but rather throughout the entire life-continuum. Second, higher EOL ambulatory and drug expenditures among socioeconomically advantaged populations irrespective of privately- or publicly-funded health care systems may underscore health service inequities that can undermine universally-funded public health care systems. SES-inequalities in health spending in universally-funded are likely smaller than those in other health care systems where affordability barriers to access medical care are more pronounced [55, 56]. Third, our results highlight the need for further research which is designed to better understand appropriateness (or lack thereof) associated with SES differences in EOL health care spending. At this time, there is limited knowledge on the cause of socioeconomic inequalities at the EOL. Specifically, further evaluation is required to better understand the health-care seeking needs of patients and the delivery-care perspectives of their providers when managing socioeconomically diverse populations. Finally, further research must not only explore whether SES-differences in EOL health care spending impacts on outcomes (e.g. quality of life), but also address the heterogeneity in methods employed across studies. This involves establishing guidelines on factors that significantly impact results, such as health care services examined, adjustment of confounders, types of SES measures.

This study had some noteworthy limitations. First, our meta-analyses combined data across studies in order to estimate the effect of SES on EOL cost; yet, socioeconomic measures, diseases, EOL time periods, and outcomes varied across studies and were reflected in the heterogeneity (I^2^). In particular, high statistical heterogeneity existed in the larger meta-analysis. In addition, the meta-analysis with adjustment for comorbidities only included two studies, which limits its statistical power. While income reflected current spending power, it may have been confounded by reverse-causality [57]. Composite measure of SES (e.g. neighbourhood income) did not permit the study of how individual SES factors impacted health, and many studies did not justify the choice of SES measure [58]. In addition, focusing on the last 12 months of life was an arbitrary choice and could only be considered a proxy for EOL cost. Lastly, there was a focus on high-income countries, which limit the applicability of conclusions of this review to middle- and low-income countries.

## Conclusions

In conclusion, our study demonstrated an association between SES was EOL health care expenditures, thereby underscoring the potential importance of SES as a determinant of health and health care delivery inequalities throughout the life-continuum. Significant heterogeneity existed in the methodology and direction of results. Overall, an inequality leading to higher end-of-life expenditure for higher SES patients exist to varying extents, even within countries providing universal health care, with greatest differences seen for outpatient and prescription drug costs. However, the directionality of such variations in SES-health-care expenditure associations may differ according to whether or not adjustment methodology is employed. Further research is needed to better understand the appropriateness of, and quality of life implications associated with such socioeconomic cost-consumption inequalities so that EOL care services can be allocated according to health needs irrespective of affordability and wealth.

## Supplementary information

**Additional file 1:.** Search Strategy.

**Additional file 2:.** Adjustments in Data Analysis.

## Data Availability

All datasets used and/or analysed during the current study are available from the corresponding author on request.

## Abbreviations

EOL: End-of-life
NHLBI: National Heart, Lung, and Blood Institute
PRISMA: Preferred Reporting Items for Systematic Reviews and Meta-Analyses
SES: Socioeconomic status
SMD: Standardized mean difference
STROBE: Strengthening the Reporting of Observational Studies in Epidemiology
USD: United States Dollars
