# Supplementary material for: Impact of socioeconomic status on end-of-life costs: a systematic review and meta-analysis
Source: BMC Palliat Care. 2020 Mar 23;19:35. doi: 10.1186/s12904-020-0538-y (PMC7087362; doi:10.1186/s12904-020-0538-y)
Supplement: Supplementary file 1 — Additional file 1:. Search Strategy. [file 12904_2020_538_MOESM1_ESM.docx]

**Additional file 1: Search Strategy**

**Ovid MEDLINE** [Searched December 2019]
<Advanced Search> [mp= title, abstract, original title, name of substance word, subject heading word, keyword heading word, protocol supplementary concept word, rare disease supplementary concept word, unique identifier] (# of studies identified)

1. Financing, government/ (20694)
2. Government* financ*.ti,ab. (339)
3. exp Medical assistance/ (60688)
4. ((medical or health) adj2 (cost* or assistance* or expenditure* or financ* or spending*)).ti,ab. (57433)
5. Health expenditure/ (19489)
6. exp resource allocation/ (16996)
7. Health care costs/ (38153)
8. exp Socioeconomic Factors/ (437223)
9. Socioeconomic factor*.ti,ab. (7241)
10. Socio-economic factor*.ti,ab. (2579)
11. (social adj2 (class* or caste* or population* or inequality*)).ti,ab. (12140)
12. (middle adj2 (class* or caste* or population*)).ti,ab. (5664)
13. (upper adj2 (class* or caste* or population*)).ti,ab. (1644)
14. (lower adj2 (class* or caste* or population*)).ti,ab. (6731)
15. High income*.ti,ab. (9574)
16. Low income*.ti,ab. (33079)
17. Inequalit*.ti,ab. (28314)
18. Povert*.ti,ab. (23804)
19. Social welfare/ (9133)
20. Social welfare*.ti,ab. (2828)
21. Social justice/ (11881)
22. Social justice*.ti,ab. (2280)
23. ((Index or indices) adj3 deprivation).ti,ab. (1646)
24. Terminal care/ (27326)
25. Terminal care*.ti,ab. (1499)
26. End of life.ti,ab. (21250)
27. End-of-life.ti,ab. (21250)
28. Last year* of life.ti,ab. (713)
29. Hospices/ (4946)
30. Hospice*.ti,ab. (11942)
31. 1 or 2 or 3 or 4 or 5 or 6 or 7 (182460)
32. 8 or 9 or 10 or 11 or 12 or 13 or 14 or 15 or 16 or 17 or 18 or 19 or 20 or 21 or 22 or 23 (517843)
33. 24 or 25 or 26 or 27 or 28 or 29 or 30 (48039)
34. 31 and 32 and 33 (215)

**Ovid EMBASE** [Searched December 2019]

<Advanced Search> [mp= title, abstract, original title, name of substance word, subject heading word, keyword heading word, protocol supplementary concept word, rare disease supplementary concept word, unique identifier] (# of studies identified)

1. Financial support.ti,ab. (6459)
2. Government* finance*.ti,ab. (116)
3. exp health care cost/ (284865)
4. exp resource allocation/ (19967)
5. ((medical or health) adj2 (cost* or assistance* or expenditure* or financ* or spending*)).ti,ab. (81083)
6. exp socioeconomics/ (379287)
7. Socioeconomic factor*.ti,ab. (9152)
8. Socio-economic factor*.ti,ab. (3418)
9. social welfare/ (19874)
10. Social welfare*.ti,ab. (3587)
11. social justice/ (10105)
12. Social justice*.ti,ab. (2410)
13. (social adj2 (class* or caste* or population* or inequality*)).ti,ab. (14782)
14. (middle adj2 (class* or caste* or population*)).ti,ab. (7329)
15. (upper adj2 (class* or caste* or population*)).ti,ab. (2303)
16. (lower adj2 (class* or caste* or population*)).ti,ab. (9330)
17. High income*.ti,ab. (11705)
18. Low income*.ti,ab. (38996)
19. Inequalit*.ti,ab. (30443)
20. Poverty.ti,ab. (28140)
21. ((Index or indices) adj3 deprivation).ti,ab. (2598)
22. terminal care/ (34833)
23. Terminal care*.ti,ab. (1856)
24. End of life.ti,ab. (30829)
25. End-of-life.ti,ab. (30829)
26. Last year* of life.ti,ab. (1010)
27. hospice/ (12706)
28. Hospice*.ti,ab. (18823)
29. 1 or 2 or 3 or 4 or 5 (350312)
30. 6 or 7 or 8 or 9 or 10 or 11 or 12 or 13 or 14 or 15 or 16 or 17 or 18 or 19 or 20 or 21 (475259)
31. 22 or 23 or 24 or 25 or 26 or 27 or 28 (65597)
32. 29 and 30 and 31 (261)

**EBSCO CINAHL** [Searched December 2019] (# of studies identified)

1. (income or socioeconomic or socio-economic or education or deprivation) AND (end-of-life or last year* of life or last month* of life) AND (cost* or expenditure* or spending*) (231)

**ProQuest** [Searched December 2019] (# of studies identified)

1. ab(income or socioeconomic or socio-economic or education or deprivation) AND ab(end-of-life or last year* of life or last month* of life) AND ab(cost* or expenditure* or spending*) (255)

**OpenGrey** [Searched December 2019] (# of studies identified)

1. (income or socioeconomic or socio-economic or education or deprivation) AND (end-of-life or last year* of life or last month* of life) AND (cost* or expenditure* or spending*) (0)

**Web of Science** [Searched December 2019] (# of studies identified)

1. (income or socioeconomic or socio-economic or education or deprivation) AND (end-of-life or last year* of life or last month* of life) AND (cost* or expenditure* or spending*) (255)
