# Supplementary material for: Impact of socioeconomic status on end-of-life costs: a systematic review and meta-analysis
Source: BMC Palliat Care. 2020 Mar 23;19:35. doi: 10.1186/s12904-020-0538-y (PMC7087362; doi:10.1186/s12904-020-0538-y)
Supplement: Supplementary file 2 — Additional file 2:. Adjustments in Data Analysis. [file 12904_2020_538_MOESM2_ESM.docx]

**Additional file 2: Adjustments in Data Analysis**

| Source | Location | SES Measured | EOL Period | Costs Measured | Results: SES-EOL Cost Relationship (Adjustment for Comorbidities) | Variables Adjusted For (Any, Including Comorbidities) |
| --- | --- | --- | --- | --- | --- | --- |
| Cunningham et al., 2011 [33] | Canada | Household income | Last year of life | Total medical cost | Negative relationship (adjusted); no relationship (not adjusted) | Health care need based on health status (aggregated diagnostic groups), age, sex, cause of death |
| Panczak et al., 2017* [30] | Switzerland | Median area-based socioeconomic position index | Last year of life | Costs covered by a public health insurance | Positive relationship (not adjusted) | None |
| Kelley et al., 2015 [25] | USA | Household wealth, individual education | Last 5 years of life | Total medical cost | No relationship (not adjusted) | None |
| Lee et al., 2015 [39] | Taiwan | Individual income | Last month of life | Costs covered by a public health insurance | Positive but not linear relationship (adjusted) | Age, gender, post-diagnosis survival time, geographic location, urbanization level of residence, status of advanced cancer, severity of comorbidity (Charlson Comorbidity Index Score) |
| Kelley et al., 2013 [27] | USA | Individual income, individual total assets | Last 5 years of life | 1 aspect of cost (out-of-pocket costs) | Positive relationship (unadjusted) | None |
| Kelley et al., 2011 [32] | USA | Individual net worth, individual education | Last 6 months of life | Medicare costs (parts a and b) | No relationship (unadjusted) | Functional status (need for assistance with activities of daily living), patient factors (age, sex, race, education, marriage, net worth, living alone, religion, Medicard, disease), region factors (referral region based on The Dartmouth Atlas of Health Care) |
| Hanratty et al., 2007* [29] | Sweden | Household income | Last year of life | Costs covered by a public health insurance | Positive relationship (not adjusted) | Age, sex, health-care utilization, major diagnostic groups (not comorbidities/health status) |
| Fahlman et al., 2006 [36] | USA | Median area household income | Last year of life | 1 aspect of cost (prescription drug costs) | Positive relationship (adjusted) | Age, gender, race, age at death, comorbidity (Charlson Comorbidity Index Score) |
| McGarry et al., 2005 [37] | USA | Household income, household wealth | Variable (last 12 months to last 3 years of life) | 1 aspect of cost (out-of-pocket costs) | Negative relationship as % of wealth (not adjusted) | None |
| Hogan et al., 2001[31] | USA | ZIP code poverty rate | Last year of life | Medicare costs (parts a and b) | Negative relationship (not adjusted) | None |
| Timmer and Kovar, 1971 [28] | USA | Household income | Last year of life | 1 aspect of cost (hospital & institutional care costs) | Positive relationship (not adjusted) | None |
| Chen et al., 2017 [35] | Taiwan | Individual income | Last month of life | 1 aspect of cost (inpatient costs) | Negative relationship (adjusted) | Patient characteristics (age, sex, comorbidities - Charlson Comorbidity Index Score, renal replacement needed, provider characteristics (age, sex, and the specialty of the primary physician, ownership and accreditation of hospital), regional characteristics (e.g. beds in region) |
| Hanchate et al., 2009 [41] | USA | Median area household income | Last 6 months of life | Medicare costs (parts a and b) | No relationship (adjusted) | Age, sex, race, total morbidity burden (Charlson comorbidity score and a Diagnostic Cost Group prospective relative risk score), cause of death, hospice use, receipt of intensive life-sustaining treatment |
| Keating et al., 2018 [42] | USA | Household income, individual education | Last month of life | Medicare costs (parts a and b) | No relationship (adjusted) | Demographic variables (age at death, year of death, urban/rural residence, sex, race/ethnicity, marital status), clinical variables (cancer type, stage at diagnosis, comorbidity - Adult Comorbidity Evaluation-27), additional analyses also included availability of services physicians’ beliefs, patients’ beliefs and supports |
| Shugarman et al., 2004* [40] | USA | Median area household income | Last 3 years of life, or year of life | Medicare costs (parts a and b) | Negative relationship for last year of life (adjusted). Positive relationship for last year of life (unadjusted). Positive relationship for last 3 years of life (regardless of adjustment) | Age, gender, race, Medicaid enrollment, rural/ urban setting, comorbidities, hospital beds, physician supply, comorbidity (Charlson Comorbidity Index Score) |
| Tanuseputro et al., 2015* [11] | Canada | Average area household income | Last year of life | Costs covered by a public health insurance | No relationship (unadjusted) | Health care utilization and cost information per use |
| Rolden et al., 2014 [26] | The Netherlands | Score based on area education and income | Last 6 months of life | Total medical cost | No relationship (unadjusted) | None |
| Murthy et al., 2017 [38] | Canada | Median area household income | Last year of life | Costs covered by a public health insurance | No relationship (adjusted) | Inflammatory bowel disease status, age, sex, residential setting, comorbidity (Aggregated Diagnostic Groups) |
| Walsh & Laudicella, 2017 [34] | England | % of area residents on government income benefits | Last 6 months of life | 1 aspect of cost (hospital costs) | Negative relationship (adjusted) | Patient factors (age at diagnosis, year of diagnosis, region, comorbidity - weighted Charlson Comorbidity Index Score), site of tumour, days from diagnosis to death |
| Menec et al., 2004 [24] | Canada | Average area household income | Last 6 months of life | Total medical cost | No relationship (unadjusted) | Age, sex, cause of death, region of residence, marital status |

* indicates study included in meta-analysis

USA = United States of America; SES = Socioeconomic status; EOL = End-of-life
